# Editorial: The four streams of the prefrontal cortex

**DOI:** 10.3389/fnana.2024.1487947

**Published:** 2024-10-16

**Authors:** Dorit Ben Shalom

**Affiliations:** School of Brain Sciences and Cognition, Ben-Gurion University of the Negev, Be'er Sheva, Israel

**Keywords:** prefrontal, motor, emotion, memory, sensory

This volume was meant to explore the narrow prefrontal model in Ben Shalom and Bonneh (2019), who proposed a model of the narrow prefrontal cortex (BA 8, BA 9, BA 10, BA 11), in terms of the four streams of information: motor (BA 8), emotion (BA 9), memory (BA 10), and sensory (BA 11).

**Figure 1:**
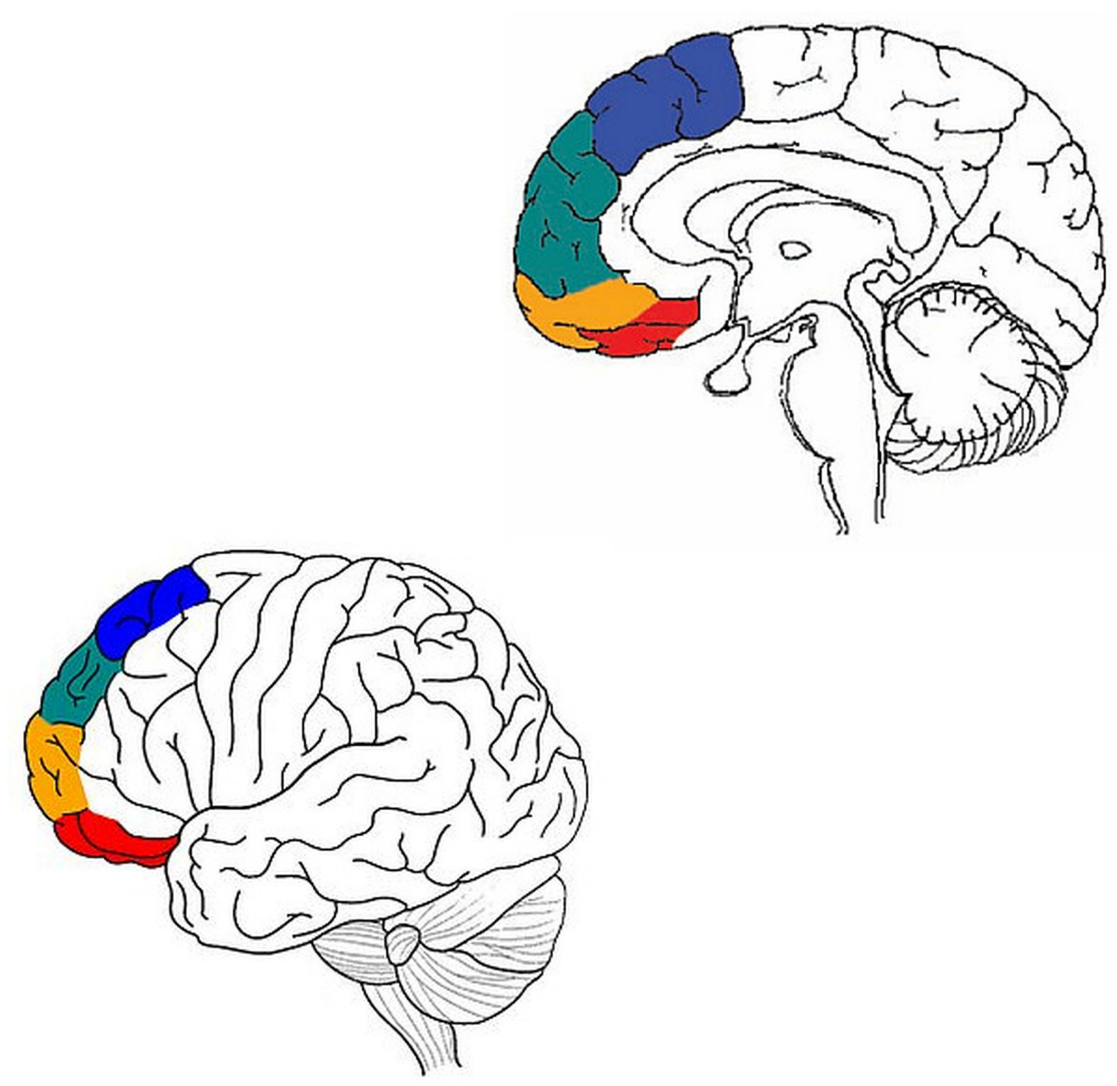
Adapted from Ben Shalom (2009) and Ronel (2018). Motor processing in blue, emotion in green, memory in orange, and sensory in red.

More specifically, starting with autism spectrum disorder (ASD), Ben Shalom (2009) conceptualized some core deficits in ASD in terms of four types of integration: medial BA 8—motor intergration; medial BA 9—emotion integration; medial BA 10—memory integration; medial BA 11—sensory integration. Ronel (2018) conducted a similar analysis for ADHD, in terms of four types of selection/inhibition: lateral BA 8—motor selection/inhibition; lateral BA 9—emotion selection/inhibition; lateral BA 10—memory selection/inhibition; lateral BA 11—sensory selection/inhibition (Figure 1).

The BA 8 (typical = human or animal with no known neurological disorder) study was authored by Dadario et al., and it concerns the motor stream, one of the four streams in the model.

Using data from the Human Connectome Project, the dorsolateral prefrontal cortex contains four subdivisions of BA8 (8BL, 8Ad, 8Av, and 8C) and two transitional areas between areas 6 and 8 (s6-8 and i6-8), while area 8BM is located in the medial prefrontal cortex (Glasser et al., 2016).

A visual inspection of the figures suggested that the two relevant subareas for the model are 8BM (medial) and 8BL (lateral). Indeed, in a study of the anatomical inputs to the sulcal portions of area 8Bm in the macaque monkey, dense labeling of cells was found in the pre-motor areas (F6 and F7) in the case with injection into the sulcal portion of area 8Bm (Eradath et al., 2015). 8BL, on the other hand, showed a high degree of functional connectivity throughout the frontal lobe, especially with other BA8 subdivisions in the dorsolateral prefrontal cortex.

The BA 9 (typical) ‘by Ben Shalom and concerns the emotion stream, one of the four streams in the model.

Smith and Lane (2015) proposed a model of emotion processing with at least three stations: areas like the amygdala that process discrete body features areas like the anterior insula that process whole-body patterns and areas like the medial prefrontal cortex that process emotion concepts. Ben Shalom and Bonneh (2019) have proposed a model of the prefrontal cortex, in which the medial BA 9 integrates emotional states, and the lateral BA 9 performs selection/inhibition on these states. Taken together, the current article suggests a pathway for emotion processing with at least four stations: areas like the amygdala that process discrete body features, areas like the anterior insula that process whole-body patterns, medial BA 9 that integrates emotion concepts, and lateral BA 9, that performs selection/inhibition on these concepts. Following the existing literature, it was then suggested that there is a significant involvement of the amygdala in psychopathy (Blair, 2008), the anterior insula in alexithymia (Bird et al., 2010), the medial BA 9 in deficits in somatosensory discrimination (Ben Shalom, 2009), and the lateral BA 9 in emotional impulsivity (Ronel, 2018).

The BA 10 (typical) article was authored by Faran. It concerns the memory stream of the four streams of the model. The study examined the involvement of BA10 in episodic memory, specifically, the predictions made by Ben Shalom and Bonneh (2019; i.e., that BA10 is involved in the integration of memory episodes) and by Ben Shalom (2009; i.e., that medial BA10 is involved in the representation of memory episodes themselves).

Based mainly on Bonasia et al. (2018), the author concluded that the association between BA10 and episodic memory indicates that incoming memory episodes are not represented in medial BA10. Instead, what is represented in medial BA10 is prior knowledge that, when activated, helps the integration of incoming episodes into prior knowledge. Thus, while there is indeed a connection between BA10 and episodic memory, as in the Ben Shalom and Bonneh (2019) model, it is not as straightforward as that of incoming memory episodes represented in medial BA10.

Leisman and Melillo provided evidence for a possible link between lateral BA 8 and motor selection/inhibition in ADHD. In their view, motor dysfunction in ADHD is a special case of an immature balance between 3 structural loops that connect the frontal cortex with the basal ganglia: the direct, indirect, and hyperdirect loops. Specifically, a relative weakness of the indirect and hyperdirect loops would result in a relative lack of the relevant functional inhibition, and in the case of motor inhibition, hyperactivity symptoms.

In terms of the four-stream model, both the direct and indirect pathways involve the promotor cortex, and thus potentially, also the pre-promotor cortex (lateral BA 8).

Sugimoto et al. provided evidence of a link between lateral BA 10 and memory selection/inhibition in ADHD.

Using a go/no-go task with a high percentage of go trials (which can arguably involve the inhibition of the relevant memory episodes), they used NIRS to measure lateral BA 10 activation in ADHD subjects during the no-go trials. Moreover, a positive correlation was observed between the right BA 10 activity and scores on Conners' Adult ADHD Rating Scales, suggesting a link between inefficiency in lateral BA 10 activation and selection/inhibition deficits in subjects with ADHD.

Segal and Elkana provided evidence of an indirect relationship between lateral BA 11, and emotionally-related sensory selection/inhibition in ADHD. Summarizing the evidence on BA 47 (which is adjacent to lateral BA 11, as opposed to BA 46, which is adjacent to lateral BA 10), they argued that the area is involved in perceptual selection that takes place through the active updating of information values linked to goal-oriented actions. In other words, while BA 46 is classically assumed to be involved in working memory for _events_, it can be argued that BA 47 is involved in working memory for emotionally relevant perceptual _objects_, thus supporting its role in the processing of emotionally relevant sensory objects.

Mohapatra and Wagner demonstrated an ambiguous and indirect connection between medial BA 9 and emotional integration in ASD. The authors described the role of two medical areas in the rodent frontal cortex, the paralimbic and infralimbic areas, in terms of socioemotional processing, including the integration of emotional states. However, they did not distinguish between the roles of the paralimbic area (arguably the rodent analog of the medial BA 9), and the infralimbic area (arguably the rodent analog of the medial BA 10; for proposed analogs of the four streams of the prefrontal cortex in the rodent brain, please see Ben Shalom and Skandalakis, 2024).

Minor et al. reviewed the evidence of a connection between the prefrontal cortex (arguably, notably medial BA 10), and memory integration in ASD. Specifically, they argued for differences in the integrity of relational memory representations and/or in the relationships between subcomponents of memory in autism.

Finally, and unexpectedly at the time, Skandalakis et al. performed population-based high-definition tractography using an averaged template generated from data from 1,065 healthy human subjects obtained from the Human Connectome Project to further elucidate the structural organization of the four streams. They reported on the structural connectivity of BA 8 with BA 6, BA 9 with the insula, BA 10 with the hippocampus, BA 11 with the temporal pole, and BA 11 with the amygdala. The four streams of the prefrontal cortex were shown to be subserved by a structural neural network that includes fibers from the anterior part of the superior longitudinal fasciculus-I and II, the corona radiata, the cingulum, the frontal aslant tract, and the uncinate fasciculus.

The full four-stream model, including both structural and functional connectivity has now been published as Ben Shalom and Skandalakis (2024).

In conclusion, this Research Topic presented functional and structural evidence for a model of the narrow prefrontal cortex (BA 8, 9, 10, and 11) in terms of four streams of information (motor, emotion, memory, and sensory, respectively). In terms of typical brain function, it is a functional neuroanatomy of the prefrontal cortex in humans, and perhaps, analogously, in mammals in general. In terms of pathological brain function, it may contribute to a better understanding of the neural circuits underlying behavioral changes in conditions such as ASD or ADHD. Future work is likely to build on this new understanding of differential information flow within the narrow prefrontal cortex.
